# Long-term effects of an alcohol prevention program at licensed premises: a Swedish 20-year follow-up study

**DOI:** 10.3389/fpubh.2024.1423708

**Published:** 2024-08-07

**Authors:** Tobias H. Elgán, Sven Andréasson, Johanna Gripenberg

**Affiliations:** ^1^STAD, Centre for Psychiatry Research, Department of Clinical Neuroscience, Stockholm Health Care Services, Karolinska Institutet, & Region Stockholm, Stockholm, Sweden; ^2^Department of Global Public Health, Centre for Psychiatry Research, Karolinska Institutet, & Region Stockholm, Stockholm, Sweden

**Keywords:** nightlife, responsible beverage service, sustainability, institutionalization, co-creation, co-production, overservice

## Abstract

**Background:**

In 1996, a multicomponent community-based alcohol prevention program in Responsible Beverage Service (RBS) targeting licensed premises was developed by STAD (Stockholm Prevents Alcohol and Drug Problems) and implemented in Stockholm, Sweden. The program consists of community mobilization and collaboration, training, and enforcement. Early evaluations have shown a significant increase in the refusal rates of alcohol service to intoxicated patrons, from 5% in 1996 to 70% in 2001, and a 29% decrease in the frequency of police-reported violence. A cost-effectiveness analysis showed a cost-saving ratio of 1:39. The program was institutionalized by a collaborative steering group consisting of community stakeholders. This study aimed to evaluate the long-term effects over 20 years of the RBS program. The indicator chosen was the rate of alcohol overserving to obviously intoxicated patrons at licensed premises in Stockholm.

**Methods:**

A 20-year follow-up study was conducted using the same procedure as the baseline and previous follow-ups. Professional male actors (pseudopatrons) were trained by an expert panel to enact a standardized scene of obvious alcohol-intoxication. In 2016, 146 licensed premises located in the central part of Stockholm were randomly selected and visited. A review of program implementation from its initiation 1996 was conducted, examining critical events, including commitment from key actors in the community, training of bar staff, and enforcement.

**Results:**

At the 20-year follow-up, pseudopatrons were refused alcohol service in 76.7% of the attempts, which was at the same level (70%) as in the follow-up in 2001, thus indicating sustained effects of the RBS program. Compared with previous follow-ups, serving staff used more active intervention techniques in 2016 toward intoxicated patrons, such as refusing to take the order (56.9% in 2016 vs. 42.0% in 2001), and fewer passive techniques, such as ignoring patrons (6.5% in 2016 vs. 15.5% in 1999) or contacting a colleague (4.1% in 2016 vs. 25% in 2001).

**Conclusion:**

The sustained long-term effects of the RBS program are unique and can be explained by the high level of institutionalization of the multicomponent program, which is still ongoing in Stockholm. These findings can inform the dissemination of the program to other countries and settings.

## 1 Introduction

A frequent observation in substance use prevention research is that research projects reporting positive outcomes in the short term often fail to find positive outcomes in the long term. Research projects are often discontinued once conclusions on outcomes have been reached; hence, long-term follow-ups spanning more than a decade are not being recorded (1). Exceptions have been found, such as in interventions aimed to prevent traffic accidents caused by driving under the influence (2–4). As such, a major challenge for substance use prevention research is to maintain interventions over time and then report on sustainable outcomes.

One high-risk environment associated with high levels of alcohol and substance use is the nightlife setting (5), which has been targeted in a number of substance use prevention interventions (6–9). Studies have shown various results but only few have been sustained over longer periods of time or have been assessed for long-term effects. In Stockholm, Sweden, a multicomponent community-based intervention was developed by the research group Stockholm Prevents Alcohol and Drug Problems (STAD) in 1996. This STAD alcohol prevention model aimed at institutionalization from its inception. Based on systems theory (10), the model sought to influence both the supply and demand systems in the nightlife context. To reproduce the intervention, the model integrated program activities into extant routines and regulations. For licensed premises, this involved changing serving practices, and for the municipal leadership and police authority, this required adopting new policies and enacting more active and coaching enforcement of rules and legislation.

Interest from stakeholder leadership was stimulated by media coverage of the research results in the early phase of the project—the very low rates of service refusal (5%) to pseudo-intoxicated patrons (11) changed to high refusal rates (70%) 5 years later (12). Similarly, the service refusal rate to pseudo-underaged patrons increased from 55 to 68% during a five-year period (13). Increased refusal rates were also accompanied by research findings of reduced rates of assaults (14) and a cost-saving ratio of 1:39 (15). This cost-saving ratio pertained to costs implementing the program and how a reduction in violent crimes translated into savings and health gains. Prior to the first study in 1996/97, the project coordinator invited relevant stakeholders in the Stockholm area to form a steering group for the project. This included the municipal licensing board, organization for restaurant owners in Sweden, police authority, county administration, and the county council. Community involvement was perceived as necessary; earlier research had demonstrated that training in responsible beverage service (RBS) in itself had a limited impact on alcohol service practices. These findings were later repeated (16, 17) and further supported in a review of RBS programs and overservice, where consumption outcomes showed mostly null results (18). Nevertheless, the programs have considerable variation in their focus and component design and delivery, and RBS may be most effectful when part of a suit of strategies delivered at the community level (6, 7, 19). Indeed, multicomponent programs combining RBS training with community mobilization, housing policies, and stricter enforcement of licensing laws have been reported to be effective in reducing violent crimes, traffic crashes, and underage sales, depending on the focus of the intervention (6, 7, 9, 20, 21).

Thus, multicomponent programs are supported in research. A key question, however, is to what extent this structure can be maintained over time, given that most community projects are short lived (1). A major challenge with STAD’s RBS program was to find ways to institutionalize it. Factors that facilitate the institutionalization of community action projects include community relevance, key leader support, co-production, indigenous staff (e.g., local residents), funding, evidence of effectiveness, and institutionalization as goal (22–24)—all of which call for policy or structural changes. Institutionalization of the RBS program required policy changes: new requirements for RBS training and more active enforcement and communication among municipal officers, the police, and licensed premises. Key indicators of institutionalization were the maintenance of a high number of trained staff every year, continuous participation from high-level representatives in regular steering group meetings and contributing resources, such as funds or staff. Structural change was indicated by written policies and regulations relevant to program activities at the organizational or municipal level. A scale measuring key factors for institutionalization—adoption, key leader support, compliance, structural changes, and sustainability—was developed by STAD. This development indicated the high degree of institutionalization of the RBS program 5 years after its inception (25).

The success of STAD’s RBS program in Stockholm led to nationwide dissemination to all counties in Sweden (26, 27). The outcomes of the dissemination were positive, even if the effect sizes were smaller than those in Stockholm, reflecting varying degrees of adherence to the model (28, 29). An attempt to replicate the model in Oslo, Norway, failed to reduce violence (30). A plausible explanation was that the project failed to reduce the rate of overserving to intoxicated patrons, a key component in the causal chain of alcohol-related violence at licensed premises. In a large European project, the STAD model was disseminated to other countries and settings (31). In the Balearic Islands, Spain, the model was pilot tested at major supermarket chains to reduce alcohol sales to underage youth. Alcohol test purchases revealed a decrease in successful alcohol sales from 77 to 46% during a two-year period, whereas the sales in a control area remained constant (32).

The RBS program has been sustained in Stockholm since its inception in 1996, and the last follow-up was recorded in 2001. The aim of the current study was to report on the outcome of a 20-year follow-up. The indicator chosen was the rate of alcohol overserving to obviously intoxicated patrons at licensed premises in Stockholm.

## 2 Materials and methods

This study conducted a 20-year follow-up to assess the sustainable effects of a multicomponent alcohol prevention intervention in the central part of Stockholm. After the baseline assessment in 1996 (11), two follow-ups had been conducted in 1999 and 2001 (12, 33).

### 2.1 Intervention

**Figure 1:**
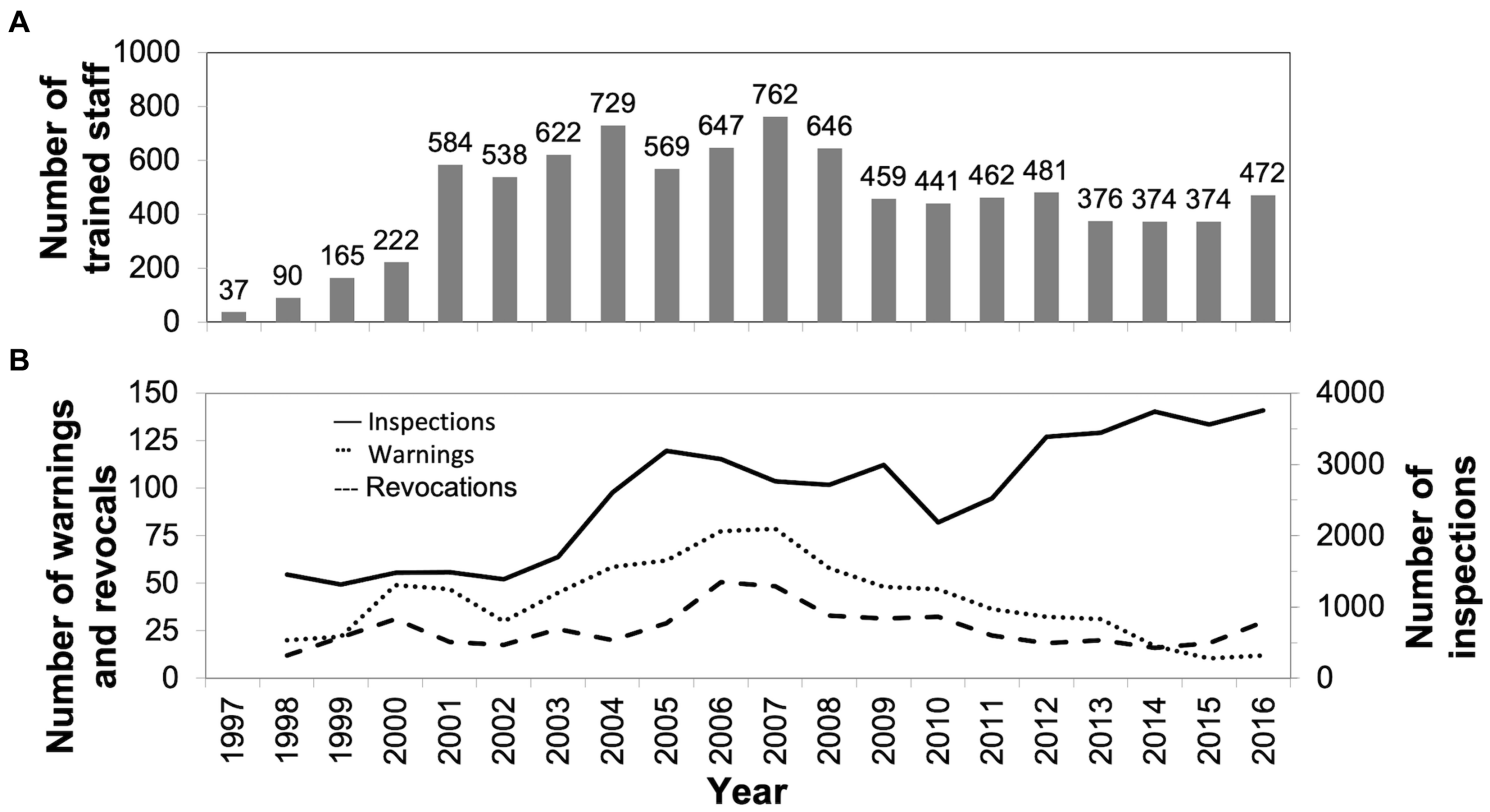
**(A)** Shows the yearly number of staff at licensed premises included in the program in Stockholm who have been trained in responsible beverage service. **(B)** Shows the number of yearly inspections (solid line), warnings (dotted line), and alcohol license revocations (broken line) issued by the licensing board in Stockholm. The lines present data based on a two-year moving average.

Strategies in STAD’s RBS program (34) were co-produced with stakeholders and include community mobilization and collaboration, training, and policy work and improved enforcement by the police authority and licensing board (Table 1). A facilitating factor that permeates most work is the co-creation of program components where stakeholders are included in a co-production process (36, 37). As of 2016, more than 9,000 staff in Stockholm had been trained (Figure 1A), and during the past 10 years, the yearly mean number of trained staff was approximately 500. Moreover, the number of yearly inspections by the licensing board in Stockholm has seen an increase from approximately 1,600 in 1997 to more than 4,000 in 2016 (Figure 1B), whereas the number of yearly revocations and warnings issued by the licensing board has remained quite constant, with a decreasing trend since the peak in 2007.

**Table 1:** Strategies of the multicomponent alcohol prevention program.

Intervention strategies	Content and dose of strategies
Community mobilization and collaboration	A baseline assessment was collected in 1996, which acted as a needs assessment demonstrating the need to mitigate alcohol service to obviously intoxicated patrons Stakeholders were identified including STAD,^a^ the licensing board at the Stockholm municipality, police authority, Public Health Agency of Sweden, Stockholm County Administrative Board, organization for restaurant owners, union for restaurant staff, and nightclub owners Great effort was put into mobilizing stakeholders using for instance results from the needs assessment and the Alcohol Act as arguments A steering group was formed after the baseline assessment with representatives from STAD, the licensing board, police authority, organization for restaurant owners, union for restaurant staff, and nightclub owners. The steering group met six times per year The steering group formulated a mutual goal with the program which was to decrease problems related to alcohol service at licensed premises In 2001, the program was considered institutionalized as all stakeholders in the steering group had agreed and signed a written agreement specifying each partner’s responsibility. This written agreement was signed by high-ranking officials from the participating stakeholders and renewed every three years When needed, working groups were formed with representatives from these organizations to co-create, for instance, training components, PR and media activities, and policy document templates Stakeholders were trained in media advocacy, which was followed by numerous contacts with journalists and editors, repeated press briefings, and publications, resulting in extensive media coverage of the program in print media as well as TV and radio. This was especially the case during the first years of the program. All media activities were conducted after prior review of materials by the collaborating partners
Training of staff at licensed premises	Two-day (eight hours each day) face-to-face training in responsible beverage service (RBS) targeting staff at licensed premises, including serving staff, doormen, and managerial staff The training, ongoing since 1997, is typically held 11 times per year. The mean number of yearly trained staff (Figure 1A) over the years is 453 Training covers topics such as medical effects of alcohol, alcohol laws and regulation, age checking, recognizing different stages of intoxication levels, conflict management, and role of enforcement officers. Lectures are held by representatives from STAD, police authority, and licensing board. The three-hour sessions on conflict management are provided by a professional actor specialized on intoxication levels in relation to alcohol service at licensed premises
Enforcement and policy work	Licensed premises were encouraged to develop alcohol policies based on policy templates, which included a strong recommendation from the licensing board to have staff at licensed premises trained In 2000, local politicians in Stockholm strongly recommended licensed premises open to 1 a.m. or later to have their staff trained The local police authority and municipal licensing board worked to enforce the Alcohol Act (35) in a cooperative manner: they coach staff and have a bidirectional conversation with staff and managers. The mean number of yearly inspections (Figure 1B) by the licensing board since 1997 was 2,580. The mean number of yearly warnings and alcohol license revocations issued by the licensing board since 1997 was 40 and 26, respectively

a A research and development unit within STAD responsible for the initiation and coordination of the intervention.

### 2.2 Design and setting of assessments in 1996, 1999, and 2001

Originally, the study was designed as a quasi-experimental control group study where the northern part of central Stockholm was the intervention area, with approximately 550 licensed premises, and the southern part was the control area, with approximately 270 licensed premises. To study overserving, the original study employed professional actors to portray a standardized scene of high level of intoxication. This method has been used by STAD (11, 12, 33, 38–40) as well as other groups (41–45) to study levels of alcohol overserving. Briefly, actors were trained by an expert panel consisting of bar staff and managers, police officers, and staff from the licensing board to enact a standardized scene of obvious alcohol intoxication. The actors were trained to act out several signs of alcohol intoxication according to a protocol before ordering a beer at the bar, such as staggering, slurred speech, and having problems focusing and fixing their gaze. The actors were also instructed to lean over the bar and fumble with the payment.

The baseline assessment in 1996 employed two male actors who were trained to conduct purchase attempts at different licensed premises (*n* = 92) during the winter. Licensed premises were selected from the county administration register to represent bars/pubs, nightclubs, hotel bars, and regular restaurants. The results revealed that the actors were refused alcohol purchase in 5% of the attempts (11, 33). Trained observers monitored all purchase attempts ensuring that the level of intoxication was the same during the attempts. They found no difference between the intervention and control areas. Following the baseline assessment, licensed premises in the intervention area were invited to have their staff undergo RBS training.

At the first follow-up during the winter of 1999, six male actors were trained and performed 103 purchase attempts. The same licensed premises as in the baseline study that were still in business (*n* = 84) were included and an additional 19 premises were added. The results revealed a statistically significant improvement compared with the baseline: the refusal rate improved from 5 to 47% (33). Notably, both study areas showed improvements in refusal rates; the two showed no difference, which was attributed to a spill-over effect given the high turnover rate of bar staff in Stockholm.

At the second follow-up during the winter of 2001, five male actors were trained and performed a total of 100 purchase attempts. The same licensed premises as in the first follow-up that were still in business (*n* = 100) were visited. The intervention was disseminated to the former control area. Results revealed an overall refusal rate of 70% (12), which was a statistically significant improvement compared with the previous assessments.

### 2.3 Design and setting of the 20-year follow up in 2016

This third follow-up was conducted during fall in 2016 using the same procedure as in the previous assessments. Because of the widespread dissemination of the RBS program in Stockholm (Table 1; Figure 1) and a requirement from the licensing board that all licensed premises that are open to 1 a.m. or later must have their staff RBS trained, we decided that a new sample of licensed premises should be selected for the study. Hence, we randomly selected licensed premises located in the central part of Stockholm from a register provided by the licensing board in Stockholm. Our initial sample included 150 licensed premises using a stratification protocol based on the closing hour—closing at 1 a.m., 3 a.m., or 5 a.m. The stratification protocol was used so that the final sample reflected the actual proportion of licensed premised in Stockholm opened to 1 a.m. (i.e., 60%) and 3 a.m. (37%). However, since they were relatively few, we included all establishments open to 5 a.m. (*n* = 17) in the study. An external researcher performed the random selection of the licensed premises.

### 2.4 Procedures

Eight professional male actors (i.e., “pseudopatrons”) between 25 and 35 years of age were recruited and trained by an expert panel to enact the standardized scene of obvious alcohol intoxication. Six observers (four males) were also recruited and trained in the procedures. The expert panel consisted of a licensed premises manager, an officer from the licensing board, a police officer, professional bartenders, a film and TV producer, and researchers from STAD (two of whom had participated in the previous assessments). The actors’ portrayal of alcohol intoxication was such that the actors should be denied service of alcohol according to the Swedish Alcohol Act (35). To ensure that the actors portrayed the same level of intoxication as in previous assessments, we filmed scenes during training sessions preceding the assessments at all time points for comparison. The scene was designed to portray an attempt to purchase a beer at a licensed establishment and was identical to the scene used in the previous studies (11, 12, 33) apart from the use of a credit card, instead of cash, as the method of payment. The actors were trained to act out several signs of intoxication according to the standardized protocol before ordering a beer, such as staggering, slurred speech, and having problems focusing and fixing their gaze. If allowed to order a beer, the actors were to insert their credit card improperly into the card machine and lean over the bar. This allowed sufficient time for the serving staff to notice the level of intoxication. All actors and observers were to sign a written non-disclosure agreement, where they agreed not to divulge their participation in the study or the results.

During a typical night of data collection, three research teams, each consisting of two actors and one observer, visited licensed premises during Wednesday, Thursday, Friday, and Saturday nights. The observer entered the establishment before the actors and sat close to the bar. Once inside the establishment, the actors approached the bar. One of them enacted the standardized scene and attempted to order a beer. The role of the “sober” actor was to interact with the other actor and to monitor how the serving staff acted. After portraying the scene, the actors left the premises followed by the observer. After each attempt, the actors and observer each completed a web-based protocol using their smartphones. The licensed premises were not informed about the visits.

### 2.5 Measures

The primary outcome was the rate of refused alcohol service to obviously alcohol-intoxicated actors attempting to order a beer at licensed premises. To record the outcomes, we developed two web-based protocols, one for the actors and one for the observers, using the survey tool Easyresearch (Questback). The actors recorded whether they were served alcohol, if the serving staff noticed the intoxication level before placing the order, and how the serving staff handled the situation (e.g., what intervention techniques were utilized if they refused service). The observers reported the serving staff’s sex and approximate age, crowdedness at the bar, overall order and intoxication level among the patrons, level of hygiene at the restrooms, music volume level, and lightning at the licensed premises. The observers had been trained to determine the different levels of these characteristics.

### 2.6 Analysis

We processed the data to generate descriptive statistics, including frequencies and proportions of denied alcohol service to “obviously alcohol-intoxicated” actors. Refusal rates are presented with 95% confidence intervals (CI). We used Fisher’s exact test to investigate statistically significant differences among the actors. Effect sizes between refusal rates were calculated using Cohen’s h where an effect size of <0.2 is considered negligible, 0.2 < 0.5 small, 0.5 < 0.8 medium, and 0.8 or more is large (46). We performed all analyses using IBM SPSS Statistics, version 23. For all analyses, an α-level < 0.05 indicated statistical significance.

## 3 Results

The teams performed 146 attempts at 146 different licensed premises. The majority of the attempts (69.2%) were performed on a Friday or Saturday, whereas 30.8% were performed on a Wednesday or Thursday. The attempts were conducted between 8:45 p.m. and 2 a.m. About a quarter of the attempts (24.7%) were conducted between 8:45 and 9:59 p.m.; 41.1% were conducted between 10 and 10:59 p.m.; 19.9% between 11 and 11:59 p.m.; and 14.4% after 12 a.m. Approximately half (50.7%) of the licensed premises were open until 1 a.m., 30.1% were open until 3 a.m., and 19.2% were open up to 5 a.m. Female and male servers comprised 29.7 and 69.0% of the total, respectively (the sex of 1.4% of the servers could not be identified). We estimated that 49.3% of the servers were 18–30 years old, 31.5% were 31–40 years old, 13.0% were 41–50 years old, and 6.2% were at least 50 years old.

**Figure 2:**
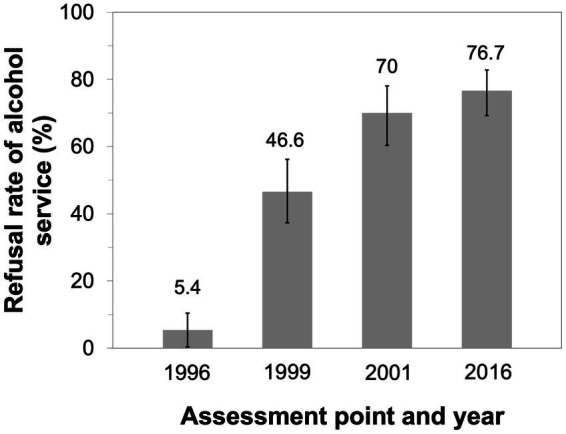
Serving staff’s refusal rates and 95% confidence intervals of alcohol service to obviously pseudo-intoxicated actors at licensed premises. Baseline assessment was conducted in 1996, and the follow-up assessments, in 1999, 2001, and 2016.

The overall refusal rate of alcohol service to obviously intoxicated pseudo-patrons was 76.7% (*n* = 112). As shown in Figure 2, the rate observed in 2016 is an improvement to the 5% in 1996 (71.3% difference, 95% CI: 58.3–84.3, Cohen’s h: 1.7) and 46.6% in 1999 (30.1% difference, 95% CI: 18.0–42.2, Cohen’s h: 0.6). The refusal rate in 2016 was not different to the 70% reported in the 2001 assessment (Cohen’s h: 0.2). Fisher’s test for independence revealed a difference between the eight actors (*p* = 0.006) in the 2016 assessment. This is explained by the two extremes where one actor was denied alcohol purchase in 48.1% of the attempts, and the other actor, in 96.3% of the attempts.

The servers used different intervention techniques to refuse the alcohol purchase of the pseudo-intoxicated actor (Table 2). The extent to which servers used different techniques varied across the assessments. The most frequently used server intervention technique in 2016 was refusing to take the order, followed by suggesting food or non-alcoholic beverage and asking the actor to leave the premises. Compared with the assessments in 1999 and 2001, servers contacted a colleague to a lesser extent in the 2016 assessment. In 2016, servers ignored the actors to a lesser extent compared with 1999 and refused to take the order to a greater extent compared with 2001.

**Table 2:** Intervention techniques used by servers across the follow-up assessments^a^ and differences between assessment points.

Server intervention	1999^b^ (*n* = 103) %	2001^b^ (*n* = 100) %	2016^c^ (*n* = 123) %	Absolute difference between proportions	
				2016–1999 (95% CI)	2016–2001 (95% CI)
Suggested food or non-alcoholic beverage	11.7	24.0	21.1	9.4 (−0.004–19.2)	2.9 (−8.1–13.9)
Ignored the patron	15.5	9.0	6.5	9.0 (0.94–17.1)*	2.5 (−4.5–9.5)
Asking the patron to leave the premises	13.6	25.0	20.3	6.7 (−3.2–16.6)	4.7 (−6.3–15.7)
Contacted a colleague	16.5	25.0	4.1	12.4 (4.6–20.2)*	20.9 (11.9–29.9)*
Refused to take the order	n.a.	42.0	56.9	n.a.	14.9 (17.0–28.1)*
Encouraged the patron to wait	0	4.0	2.4	2.4 (−0.01–5.4)	1.6 (−3.0–6.2)

a The refusal rate at baseline in 1996 was 5%; data on intervention techniques were not included.

b Results from follow-up assessments in 1999 and 2001 have been presented previously (12, 33).

c Missing data in 2016 assessment, *n* = 23.

*Statistically significant differences between proportions.

## 4 Discussion

This study examined the sustainable effects of a multicomponent community-based alcohol prevention intervention implemented in Stockholm. Results from the 20-year follow-up demonstrated sustained effects—the refusal rate of alcohol service to obviously alcohol-intoxicated patrons at licensed premises was 77%, the same level as in the follow-up assessment 15 years ago (12). Compared with the 5% refusal rate observed at the baseline assessment in 1996 (11), the rate in this study is a large improvement as indicated by the large effect size. In a comprehensive review, Babor et al. (8) note that conclusions from community-based prevention research are mixed, but that overall, small effects are found. While there are exceptions to these findings, these suffer from sustainability problems where positive results have not been maintained over time. Based on the first five-year follow-ups, Babor et al. conclude that STAD’s RBS program stands out as an exception where results were maintained over time. The sustained long-term effects can be attributed to the overall strategies used in the multicomponent RBS program: community mobilization and collaboration, training, and enforcement, all of which have been implemented in collaboration with stakeholders. The significance of training is indicated by the different intervention techniques used by serving staff to refuse obviously intoxicated patrons. An important component of the two-day RBS training is the three-hour session on conflict management where active intervention techniques are taught and practiced. Indeed, our current results demonstrate that serving staff more frequently used active intervention techniques, such as refusing patrons, suggesting food or non-alcoholic beverage, or asking patrons to leave the premises. At the baseline assessment in 1999, passive techniques, such as ignoring patrons, were more frequently employed. Further, in the 2016 assessment, serving staff approached by the pseudo-intoxicated actor contacted a colleague to a lesser extent compared with the two previous assessments. This may be attributed to the training that has increased serving staff’s capacity to proactively intervene with patrons who are obviously intoxicated, which has been observed in other studies (1).

The facilitating factors for the sustained effects of programs are their status of being mandatory and institutionalized (1, 22, 47, 48). In 2000, local politicians in Stockholm strongly recommended RBS training for staff at licensed premises with opening hours to 1 a.m. or later (25). A high level of institutionalization of STAD’s RBS program in Stockholm was also reported 5 years after the program’s inception (25). Our current results confirmed the high level of institutionalization, further supported by several observations. The yearly number of trained staff in Stockholm was high; the mean number of yearly trained staff during the past 10 years was approximately 500. The number of inspections by the licensing board saw an increase during the past 10 years, whereas the number of warnings decreased during the same period. Thus, training and enforcement may have resulted in a higher level of compliance by licensed premises to the alcohol law. Further, a system for collaboration was developed involving representatives from STAD, the licensing board, police authority, organization for restaurant owners, union for restaurant staff, and nightclub owners who met in six steering group meetings per year. Each partner’s responsibility in this collaboration was specified in a written agreement in 2001, signed by high-ranking officials and renewed every 3 years. By signing a well-conceived written agreement, the organizations and authorities guarantee the continuation of the program, where activities are not dependent on specific individuals but instead are integrated in extant routines and regulations (25, 49). When needed, representatives from this action group have participated in co-creating key intervention components, such as training, and media activities, without any external funding provided. Co-creation has been a contributing factor to the formation of a sense of intervention ownership among stakeholders and is a facilitating factor for successful implementation and sustainability (36, 37).

In the alcohol policy literature, the most effective policies are those that reduce the economic and physical availability of alcohol (8, 50, 51). These include regulating the density of alcohol outlets (i.e., shops and licensed premises) as well as regulating business hours. Even when these policies are implemented, alcohol-related problems will still occur, thereby highlighting the need for effective programs that can minimize these problems. Our study demonstrated the long-term effectiveness of one such program and illustrated the results that can be achieved by implementing a multicomponent program with strategies such as collaboration, training, and enforcement. These strategies can also be applied successfully to other settings and substances. For instance, in 2015 to 2017, a project was conducted at large sporting events in Stockholm with the aim to decrease intoxication levels and increase refusal rates of obviously intoxicated spectators (39, 52). The RBS program was subsequently tailored and implemented at two sport arenas (hosting up to 30,000 and 50,000 spectators, respectively) close to the Stockholm city centre (38, 53). The baseline refusal rate at licensed premises adjacent to the arenas was 68%, and that at licensed premises inside the arenas was 32% (38). This discrepancy was explained by staff at premises outside arenas having been RBS trained, whereas staff at premises inside the arenas had not. The tailored RBS program was then implemented at the arenas and the two-year follow-up assessment revealed that the rate at premises inside arenas had improved to 57% and the rate outside arenas remained at 73%. Yet another program utilizing the same core components as in the RBS program is the Clubs against Drugs program targeting substance use in the nightlife context. The implementation of this program also resulted in an increase in staff intervention toward intoxicated pseudo-patrons (54), which was further improved in a five-year follow-up (55). A decrease in staff’s own substance use and observed substance use among patrons was also seen over this time period (56). The football project and the Clubs against Drugs program both illustrate that the RBS program can be tailored to other settings and substances. The aforementioned European project further demonstrated that the program can be disseminated to other countries and yet another setting—supermarket chains—to reduce alcohol sales to underaged youth (31, 32).

### 4.1 Strengths and limitations

The strengths of the study included the long-term assessment of an ongoing program that has been institutionalized and sustained in Stockholm over 20 years. For the sake of reliability, each attempt by the pseudo-intoxicated actor was monitored by an observer who ensured the actors’ conformance to the protocol and portrayal of the correct level of intoxication. The level of intoxication was also the same as in previous STAD studies (12, 33, 38, 39). The calibration of the correct intoxication level was facilitated by footage from actor training sessions in previous assessments and the fact that two of the authors had participated in previous assessments. Eight actors were employed to mitigate the possible effects of any individual-level characteristics. We also analyzed records on intervention dosage regarding collaborative meetings, training, and enforcement by the licensing board.

Meanwhile, the limitations of our study included the absence of a control area and the use of a cross-sectional design, both of which limit conclusions on causality. However, maintaining a control area over 20 years was not feasible and many of the licensed premises included in the original study were no longer in business. Moreover, since 2000, staff at all licensed premises in Stockholm open up to 1 a.m. or later have undergone mandatory training (25). As such, no control area could be designated in Stockholm. Further, we could not control for the RBS training of the serving staff tested, as this was a covert study. Over the years, more than 9,000 serving staff in Stockholm have been trained. Another limitation was in the differences among the eight actors in terms of refusal rates. All actors had been trained by an expert panel and each attempt was observed by a research assistant who gave feedback on the level of intoxication. The discrepancy in refusal rates indicates the impacts of other individual-level factors, such as physical appearance. The use of only male actors was also a limitation. This was in line with the previous assessments where only males had been used (12, 33). Meanwhile, despite having the police authority as a collaborating partner in the project, we could not retrieve records on enforcement by the police. Finally, the study took place in Stockholm, which limits generalizability to other cities and countries.

## 5 Conclusion

Results from this novel 20-year follow-up assessment demonstrated that the refusal rate of alcohol service to obviously alcohol intoxicated patrons at licensed premises in Stockholm remained at the same high level as in the last assessment 15 years ago. This can most likely be attributed to sustained effects of STAD’s RBS program, including stakeholder mobilization and collaboration, training of staff, and enforcement by the licensing board and police authority. The program has been ongoing in Stockholm since 1996 and was institutionalized in 2001 with written agreements, mandated training, and a continued focus on co-creation which facilitates a sense of ownership. Our findings can inform the dissemination of the program to other countries and settings.

## Data availability statement

The raw data supporting the conclusions of this article are not publicly available. The data are available from the authors upon reasonable request and with permission from the Centre for Psychiatry Research.

## Author contributions

TE: Conceptualization, Data curation, Formal analysis, Investigation, Methodology, Writing – original draft, Writing – review & editing. SA: Conceptualization, Methodology, Writing – original draft, Writing – review & editing. JG: Conceptualization, Data curation, Funding acquisition, Investigation, Methodology, Supervision, Writing – review & editing.
